# A novel pH sensitive theranostic PLGA nanoparticle for boron neutron capture therapy in mesothelioma treatment

**DOI:** 10.1038/s41598-023-27625-0

**Published:** 2023-01-12

**Authors:** Jacopo Sforzi, Alberto Lanfranco, Rachele Stefania, Diego Alberti, Valeria Bitonto, Stefano Parisotto, Polyssena Renzi, Nicoletta Protti, Saverio Altieri, Annamaria Deagostino, Simonetta Geninatti Crich

**Affiliations:** 1 Department of Molecular Biotechnology and Health Sciences, University of Torino, Via Nizza 52, 10126 Turin, Italy; 2 Department of Chemistry, University of Torino, Via P. Giuria 7, 10125 Turin, Italy; 3 Department of Science and Technological Innovation, Università del Piemonte Orientale, 15121 Alessandria, Italy; 4 Department of Physics, University of Pavia, Via Agostino Bassi 6, 27100 Pavia, Italy; 5 Nuclear Physics National Institute (INFN), Unit of Pavia, Via Agostino Bassi 6, 27100 Pavia, Italy

**Keywords:** Cancer, Drug discovery, Molecular medicine, Chemistry

## Abstract

This study aims to develop poly lactic-co-glycolic acid (PLGA) nanoparticles with an innovative imaging-guided approach based on Boron Neutron Capture Therapy for the treatment of mesothelioma. The herein-reported results demonstrate that PLGA nanoparticles incorporating oligo-histidine chains and the dual Gd/B theranostic agent AT101 can successfully be exploited to deliver a therapeutic dose of boron to mesothelioma cells, significantly higher than in healthy mesothelial cells as assessed by ICP-MS and MRI. The selective release is pH responsive taking advantage of the slightly acidic pH of the tumour extracellular environment and triggered by the protonation of imidazole groups of histidine. After irradiation with thermal neutrons, tumoral and healthy cells survival and clonogenic ability were evaluated. Obtained results appear very promising, providing patients affected by this rare disease with an improved therapeutic option, exploiting PLGA nanoparticles.

## Introduction

**Scheme 1:**
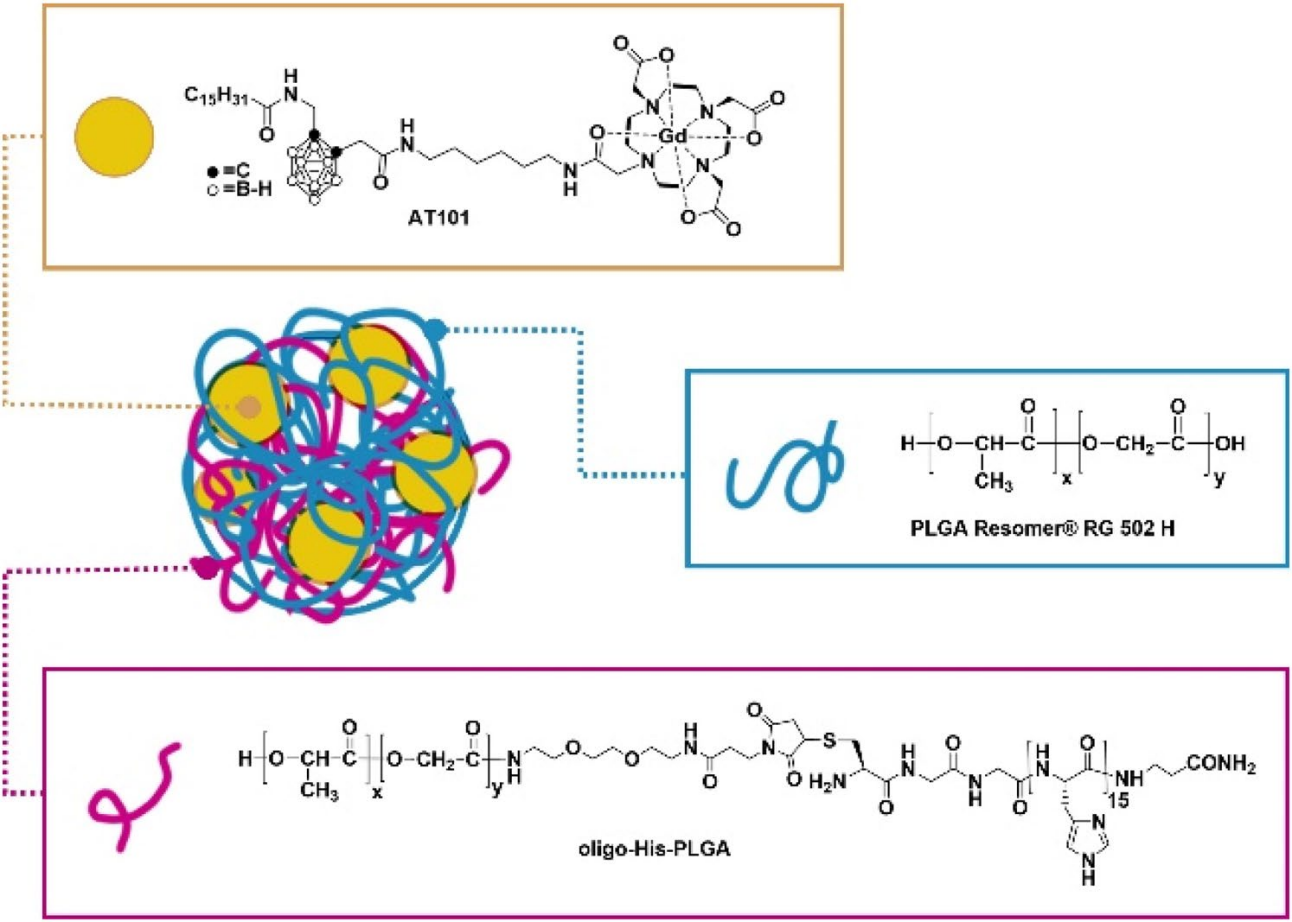
Schematic representation of PLGA-His nanoparticles.

Malignant mesothelioma (MM) is an aggressive tumour with a poor prognosis whose incidence and mortality are a function of past asbestos exposure, even after a latency period of 30–50 years. MM is recognized as a rare occupational disease lacking any effective therapy, and the median survival after diagnosis is less than 9–12 months^1,2^. MM is a disseminated tumour spreading inside the whole pleura or peritoneum. Conventional radiotherapies have limited effectiveness due to several radiosensitive tissues, which limit the maximum dose deliverable to the malignant nodules. Nowadays, targeted therapies represent one of the major focuses of cancer research, and many future advances in cancer treatment are expected to come from this approach. However, an effective therapy based on molecular targeting against MM does not exist yet. It remains, basically, a challenge for clinicians to perform an early MM diagnosis because of the lack of knowledge of precise MM biomarkers. Despite the numerous studies aimed at finding suitable biomarkers in blood and pleural effusions, these efforts have not yet produced an effective diagnostic tool^3,4^. Therefore, the standard treatment option remains an invasive biopsy followed by a chemotherapy with *cisplatin* and Pemetrexed, with or without Bevacizumab^5^, with many side effects and low efficacy. In this context, polymeric nanoparticles can be a good option for the delivery of drugs and imaging agents to mesothelioma cancer cells, improving therapeutic and diagnostic efficacy and decreasing off-target toxicity^6^. Nanoparticles can specifically release drugs at the tumour site due to the local enhanced permeability and retention effect. Among different biodegradable polymers, that recently have received great attention, poly lactic-co-glycolic acid nanoparticles (PLGA-NPs) have been proposed as delivery agents for cancer treatment^7–10^. Between the US FDA approved drug delivery systems, PLGAs are some of the most effective biodegradable polymers, due to their controlled and sustained-release properties, low toxicity, and biocompatibility with tissue and cells. In this paper, we loaded the PLGA-NPs with a dual therapy-diagnostic compound carrying a Gd-based magnetic resonance Imaging (MRI) contrast agent and a carborane moiety (icosahedral lipophilic clusters containing boron atoms) used for Neutron Capture Therapy (NCT). NCT is an example of targeted therapy with good efficacy and low toxicity that provides a tumour-selective cell death^11–13^. More specifically, this therapy can combine low-energy thermal neutron irradiation with the presence of boron-containing agents in the targeted pathological tissues. Neutrons are captured by non-radioactive ^10^B, triggering a decaying nuclear reaction releasing alpha particles and ^7^Li, causing a high biological damage in approximately 10 µm of diameter, which is less than the average diameter of a mammalian cell. Therefore, administering boron and producing alpha radiation selectively in diseased cells, NCT can kill the pathological ones while sparing the surrounding healthy tissues. These characteristics make BNCT a promising treatment for diffused metastasis and infiltrating tumours such as mesothelioma, that cannot be treated or are resistant to methods usually applicable in a localized tumour mass, such as conventional radiotherapy or surgery^14^. BNCT has been applied to skin melanoma, brain, head and neck tumours, and a large amount of clinical data has been collected through the different clinical trials (I/II phase) carried out in Japan, USA, Netherlands, Sweden, Finland, Argentina, and Taiwan^15,16^. Nakamura and co-workers recently developed a hyaluronic acid containing sodium mercaptoundecahydro-closo-dodecaborate (BSH) specifically delivered to MM in preclinical mouse models^17^. Moreover, in 2006, a small number of MM patients were safely treated with BNCT in Japan, achieving significant palliation of the symptoms^18^. However, to achieve BNCT efficacy, the boron carriers have to respect different conditions: (i) low systemic toxicity; (ii) high selectivity for tumour cells; (iii) long half-life within the tumour for the duration of the treatment. It has been estimated that approximately 10–30 μg of B per gram of tumour mass are needed to attain an effective treatment, using a tolerable irradiation time and a suitable neutron source^19^. The two compounds currently used in clinical trials are *p*-borono-l-phenylalanine (BPA) (used in trials for glioblastoma, head and neck cancer, and melanoma) and BSH (designed for brain tumour treatments). These agents yield a tumour to normal tissue boron concentration ratio between 3 and 6, allowing a safe and quite effective treatment. However, it is a widespread opinion in the scientific community that a wider clinical application of BNCT will be possible when the uptake in targeting tumour cells will be further improved^19^. As an example of a successful targeting strategy, Low Density Lipoproteins (LDL) loaded with AT101, have been proposed by our group as an endogenous lipid carrier for the specific delivery of boron to different kind of tumours such as melanoma^20^, pulmonary breast metastases^21^ and recently mesothelioma^22^. AT101 (Scheme 1) is a dual agent containing a carborane moiety functionalised with a Gd-based contrast agent. Among boron derivatives, carboranes occupy a special position both for their high boron content (10 B atoms) and their chemical versatility coupled with a high in vivo stability^23–26^.

In this study, PLGA nanoparticles functionalized with unique amino acid sequence consisting of 15 histidine (His) residues (CGGH_15_βA), namely PLGA-His, have been developed for the controlled release of the B-containing agent in the proximity of the tumour microenvironment with a pH triggered mechanism. In fact, the pKa of histidine in its oligomeric form is approximately 6.8^27^, thus being below pH 7 the imidazole ring is protonated. This occurs also when the oligo-His is conjugated to a PLGA polymeric chain forming a stable nanoparticle^28^. This was demonstrated thanks to the presence of ^14^N atoms of histidine causing a quadrupolar relaxation enhancement (also called Quadrupolar Peak, QP) at 1.39 MHz, well detectable using a Fast Field Cycling NMR relaxometer^28^. The relaxation enhancement is pH dependent in the range 6.5–7.5, thus it acts as a reporter of the nanoparticle or scaffold integrity^28,29^. As it has been demonstrated that in the cancer microenvironment the pH is altered and more acidic than the physiological one^30^, herein, we propose the use of these B and Gd containing pH-sensitive nanoparticles which selectively interact with cell membranes to release the encapsulated drug in the proximity of tumour tissues. The use of poly-histidine conjugated polymers hybrid materials for pH responsive doxorubicin delivery are already reported in the literature^31,32^. Moreover, in this study, the pH-triggered selective delivery of B and Gd containing theranostic NPs was used for a selective mesothelioma treatment with BNCT. The presence of Gd-based MRI reporters^33^ allows the indirect quantitative determination of boron at the target site before and during neutron irradiation^33–36^. This approach may open new therapeutic strategies for patients affected by MM, leading to better treatment outcomes in terms of survival time and quality of life.

## Methods and materials

PLGA Resomer^®^ RG 502 H, Mowiol 4–88 (Poly(vinyl alcohol), Mw ~ 31,000), DSPE-PEG(2000)methoxy(1,2-distearoyl-sn-glycero-3-phosphoethanolamine-N[methoxy(polyethylene glycol)-2000] and all other chemicals and solvents were purchased from Merk Life Science S.r.l. (Milano, Italy).

**Oligo-His-PLGA** was prepared according to the previously reported procedure^28^. Briefly, peptide sequence CGGH_15_βA was assembled by automated solid phase peptide synthesis and then conjugated to a PLGA-PEG_2_-Mal, prepared in-house from PLGA Resomer^®^ RG 502 H and Maleimide-PEG_2_-NH_2_. ^**10**^**B enriched AT101** (^10^B enriched ligand-C-[N-(DOTAMA-C6)-carbamoylmethyl]C′-palmitamidomethyl-o-carborane) was prepared according to the previously reported procedure^37^.

### Nanoparticles preparation

PLGA NPs were obtained following the o/w emulsion method^38^. The organic phase was prepared by dissolving 12.5 mg of PLGA Resomer^®^ RG 502 H, 12.5 mg of Oligo-His-PLGA, 2.2 mg of DSPE-PEG(2000)methoxy and 3 mg of AT101 dissolved in 0.5 ml of 3:1 (v/v) chloroform–methanol. The PLGA-CTRL was made following the same procedure by dissolving 25 mg of PLGA RG 502H, without any part of the -Oligo-Histidine conjugated Resomer. The water phase consisted of 3 ml of a 3% (w/v) Mowiol 4–88 aqueous solution. The organic PLGA solution was mixed with the PVA solution drop by drop and immediately sonicated at 4 °C, 4 times, for 1 min, at 100% sonication power. The organic phase was removed via rotary evaporation in a 500 ml glass round flask for 2 h. After the evaporation, the untrapped drug was removed by dialysis (molecular weight cut-off 14,000 Da) at 4 °C in 2 L of isotonic NaCl/Hepes buffer (HBS). The excess of PVA was removed by washing the NPs-solution with a vivaspin 20 filter (Sartorius AG, Goettingen, Germany, cut-off of 1 × 10^6^ Da) and at the end of the washing step, a final volume of 3 mL was reached for all the nanoparticles suspensions considered. Gd and B amount incorporated in PLGA nanoparticles were measured by inductively coupled plasma mass spectrometry (ICP-MS; element-2; Thermo-Finnigan, Rodano (MI), Italy) after sample digestion performed with concentrated HNO_3_ (70%, 0.4 mL) by heating at 150 °C for 8′ under microwave digestion system (ETHOS UP Milestone, Bergamo, Italy). The amount of Gd was double-checked by ^1^H nuclear magnetic resonance R_1_ measurement at 21.5 MHz, 25 °C (Stelar Spinmaster, Mede, Italy) of the mineralized complex solution (in 6 M HCl at 120 °C for 16 h). The hydrated mean diameter and z-potential of nanoparticles was determined using a dynamic light scattering (DLS) Malvern Zeta sizer 3000HS. (Malvern, UK) Nanoparticles were stored in the dark at 4 °C until further analysis.

### Stability of PLGA-CTRL and PLGA-His NPs

The stability of PLGA-CTRL and PLGA-His NPs was performed by incubating both the NPs in 40 mL of NaCl/Hepes buffer at pH 6.0 or 7.4 at 37 °C under dialysis in a 14 KDa membranes and their longitudinal relaxation rates (R_1 obs_) were measured after 3, 6, 24, 48 and 72 h at 21.5 MHz and 25 °C.

### Cell lines

AB22 murine mesothelioma and MeT-5A human healthy mesothelial cell lines were purchased from Sigma and ATCC respectively. AB22 cells were cultured in RPMI media supplemented with 25 mM Hepes, 10% FBS, 1% P/S and 2 mM of Glutamine, while MeT-5A were cultured in Medium 199 supplemented with 10% FBS, 1% P/S, 8.7E^−4^ mM of bovine insulin, 3.3E^−6^ mM of human EGF, 4E^−4^ mM of hydrocortisone and Trace Elements B (Corning) and maintained in a 5% CO_2_ incubator at 37 °C.

### Cell uptake

5 × 10^5^ AB22 and MeT-5A cells were seeded in a 6 cm diameter petri dishes and maintained until 80% of confluence were reached. Subsequently, increasing concentrations of Gd-containing PLGA-CTRL and PLGA-His nanoparticles were incubated for 24 h at 37 °C. After incubation, the cells were washed three times with PBS and detached with a solution of Tripsin/EDTA (Ethylenediaminetetraacetic acid). Then the cell pellets were resuspended in 0.2 ml PBS, sonicated (30% power, 30 s) in ice and mineralised according the previously reported procedure. Cell proteins of each sample were quantified using the commercial Bradford assay (Biorad). The nmoles of Gd and B internalized by each cell sample, measured by ICP-MS, were normalized to the total cell number. The cell number was obtained from the mg of cell proteins measured by Bradford assay, using the calibration curve: [(mg protein)/(number of cells)]. From this calibration 1 million of AB22 and MeT-5A correspond to 0.606 and 0.435 mg of proteins, respectively. From ICP-MS data, it was possible to calculate boron ppm by assuming a density of ca. 10^8^ cells for cm^3^ in the case of epithelial tumours (with a diameter ranging 15–20 µm)^38^.

### MRI

T_1_ weighted images: glass capillaries containing cells pellets of untreated or treated with NPs (0.0974 mM [Gd]) AB22 and MeT-5A were placed in an agar phantom and acquired by MRI using a Bruker Avance Neo 300 MHz spectrometer (7 T) equipped with a Micro 2.5 microimaging probe (BrukerBioSpin, Ettlingen, Germany). T_1W_ images were obtained using a standard MSME (Multi-Slice Multi-Echo) sequence with the following parameters: TR = 250 ms, TE = 3 ms, FOV = 12 × 12 mm, slice thickness = 1 mm, NEX = 6, matrix size 128 × 128.

### Neutron irradiation

T25 flasks of AB22 and MeT-5A were treated for 24 h in the presence of PLGA-CTRL and PLGA-His NPs (97 µM [Gd] and 970 μM [B]). After the incubation, cells were washed with PBS and their medium was renewed. The treated flasks and flasks containing non-treated control cells were irradiated for 15′ at 30 kW reactor power in the thermal column of the TRIGA Mark II reactor at the University of Pavia, Italy. At the end of the irradiation, the medium was removed, it was replaced with fresh medium and flasks were placed at 37 °C in a humidified atmosphere of 5% CO_2_ for other 24 h. The day after, the untreated and the treated flasks were detached with Trypsin/EDTA and the trypan blue exclusion assay was performed. The viability of irradiated untreated and treated cells were compared with not irradiated ones. The percentage of viable cells of each condition was calculated by setting the number of untreated not irradiated cells at 100% viability.

### Clonogenic assay

Clonogenic assay of irradiated and non-irradiated treated and untreated cells was performed seeding around 200 cells from each different flask in 6 cm diameter culture dishes. The growing of cells was followed for 18 days. The medium was renewed after 2–3 days. After 18 days, the cells were washed with PBS, fixed in methanol (25′) and stained with 0.5% (w/v) Crystal Violet in 20% ethanol (30′). The crystal violet was removed carefully and rinsed with tap water. Finally, the number of colonies was calculated by ImageJ software. The % was calculated by setting the irradiated control cells to 100%.

## Results and discussion

### PLGA nanoparticles preparation and characterisation.

The conjugation of PLGA with the oligo-His (Oligo-His-PLGA) was carried out following an already reported procedure^28^, based on a thiol-Michael addition reaction between PLGA-PEG_2_-maleimide and an oligo-His containing a *N*-terminal cysteine residue (amino acid sequence: CGGHnβA, n = 15). PLGA-NPs were prepared following the o/w emulsion solvent extraction method^38^ using 100% of the commercially available PLGA (MW = 7–17 kDa; named PLGA-CTRL) or mixing 50% of this PLGA with 50% of Oligo-His-PLGA (PLGA-His). The theranostic agent AT101 (Scheme 1) was loaded into the NPs by adding it to the organic phase containing PLGA polymers. After dialysis the amount of AT101 remaining encapsulated with a very good yields (> 82%), was determined by measuring both B and Gd by ICP-MS. The average hydrodynamic diameters of PLGA nanoparticles, the millimolar relaxivity and the average surface-potential of the nanoparticles are reported in Table 1.

**Table 1:** Characterization of PLGA-His and PLGA CTRL NPs.

	PLGA-His	PLGA-CTRL
**Size (nm)**	142.7 ± 4.2	153.5 ± 3,2
**Loading yield (%)**	84	82
**PDI (polydispersion index)**	0.139 ± 0.01	0.146 ± 0.002
**r**_**1p**_** = (R**_**1obs**_**-0.38)/[Gd]mM**	18.1 ± 1.4	18.0 ± 1.1
**Surface Z potential (mV)**		
pH 7.36	− 2.80 ± 0.2	− 3.5 ± 0.6
pH 6.5	− 2.06 ± 0.1	− 3.41 ± 0.2
pH 5.0	− 2.10 ± 0.2	− 3.33 ± 0.5

The low and slightly negative Z potential observed in both NPs is due to the shielding effect of the negative charges of PLGA caused by both PVA and DSPE-PEG coating^39,40^. For the same reason only a slight Z potential increase was measured on PLGA-His at pH < 6.5. Moreover, part of the protonated oligo-His may remain in the particle core thus reducing their contribution to the measured Z potential (Table 1). The nanoparticles were stored at 4 °C and their size was measured by DLS every 15 days. The results are reported in Table 2.

**Table 2:** PLGA-CTRL and PLGA-His stability at 4 °C.

NP	Day 0	Day 14	Day 30
PLGA-CTRL size (nm)/PDI	153.5 ± 3.2/0.15	156 ± 3.0/0.15	158.6 ± 4/0.18
PLGA-His size (nm)/PDI	142.7 ± 4.2/0.14	143.8 ± 2.8/0.15	149.0 ± 3.8/0.18

**Figure 1:**
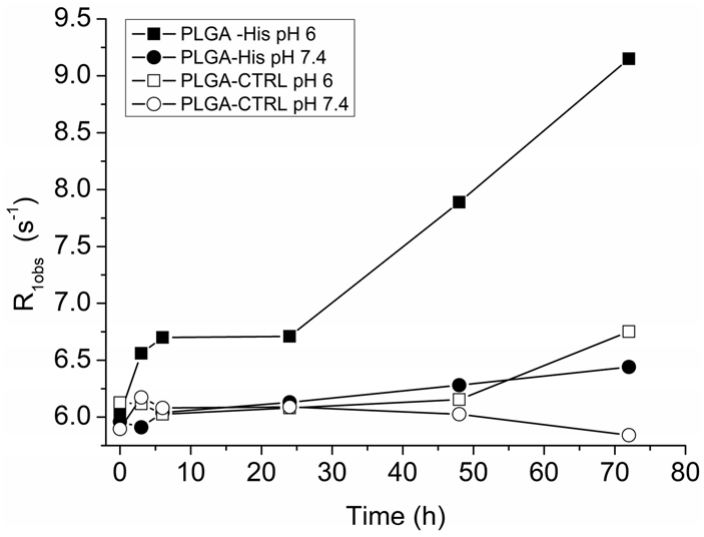
Longitudinal relaxation rates (R_1_, s^−1^) of HBS solutions maintained at 37 °C for maximum 72 h containing PLGA-CTRL at pH = 7.4 (O) and pH = 6 (filled circle) or PLGA-His at pH = 7.4 (open square) and pH = 6 (filled square).

The relaxivity (r_1p_) is an expression of the contrast agent efficiency and corresponds to the paramagnetic contribution to the longitudinal relaxation rate normalized to the mM concentration of the Gd-complex solution. Both PLGA-CTRL and PLGA-His show a 4–5 times higher r_1p_ value (ca. 18 s^−1^ mM^−1^, at 21.5 MHz and 25 °C, Table 1) with respect to those one observed with clinically used Gd-based contrast agents (in the range 4–5 mM^−1^ s^−1^)^41^. This is an indication of the long tumbling time filled by the AT101 complexes when loaded in the NP and the enhanced sensitivity reduces the detection limit of these imaging probes when internalized in a NP. The stability at 37 °C of the delivery system was assessed by monitoring for 72 h the relaxation rates of PLGA-NPs solutions at both physiological and tumoral pH, in a saline buffer (Hepes-NaCl buffer). To this purpose, solutions containing PLGA-CTRL and PLGA-His were stirred at 37 °C, both at pH 7.4 and 6.0, for 72 h under dialysis (cut-off 14 KDa). The relaxation rates of the PLGA solution in the dialysis membrane were measured after 3, 6, 24, 48, and 72 h of incubation. Despite Fig. 1 shows that PLGA-CTRL are essentially stable for 72 h in these conditions, in the case of PLGA-His, there was observed an increased R_1_ (s^−1^) when incubated at acidic pH, due to the protonation of the imidazole groups present in the NPs, with a consequent alteration of its physicochemical structure producing a swelling of the NP that facilitates water permeation. The swelling of the NP was confirmed by measuring the size of both PLGA-CTRL and PLGA-His after 24 h incubation at pH = 6.0 and 37 °C. In fact, despite the hydrodynamic diameter measured on PLGA-CTRL remained almost unchanged (149.0 ± 4 nm) the measured diameter of PLGA-His at pH 6.0 (160.4 ± 3.2 nm) increased of about 12% thus confirming the nanoparticle swelling and increase in volume. Moreover, as reported in the introduction oligo-histidine chains show a characteristic relaxation peak at 1.38 MHz due to the ^14^N nuclear quadrupole resonance frequency of the imidazole groups present in the oligomeric chain, well distinct from the QPs generated by the background tissue. As the ^14^N Quadrupolar Relaxation peak may be only observed for immobilized systems, changes in the intensity of the imidazole QPs can act as a reporter of the physical state of the poly-His solid/ liquid status and swelling related to the pH of the microenvironment. In reference 28 it was shown that the protonation of the imidazole group of the histidine (pKa = 6.8) increased the particle swelling and polymer solubility with a consequent increase in mobility and progressive disappearance of the QPs at pH < 6.0. Unfortunately, the QPs were not detectable in PLGA-His used in this work as the loaded paramagnetic Gd complex increases dramatically the relaxation rate becoming the predominant relaxation process.

In order to confirm this hypothesis, the Gd concentration was measured in the solutions inside the dialysis membrane (diluted 1:1 with buffer) after 0, 24 and 72 h incubation at 37 °C at both pH 7.4 and 6. The results reported in Table 3 shows that only at pH 6 a significant AT101 release was observed after 24 and 72 h incubation thus confirming the reduced stability of the nanoparticle at this pH.

**Table 3:** NP stability and AT101 release at pH 7.4 and 6.

NP	pH	Gd mM 0 h	Gd mM 24 h	Gd mM 72 h	% release 24 h	% release 72 h
PLGA-CTRL	7.4	0.154 ± 0.007	0.151 ± 0.006	0.149 ± 0.006	2	3
PLGA-CTRL	6.0	0.155 ± 0.005	0.150 ± 0.005	0.148 ± 0.005	3	4
PLGA-His	7.4	0.155 ± 0.004	0.150 ± 0.005	0.146 ± 0.005	3	6
PLGA-His	6.0	0.156 ± 0.006	0.141 ± 0.006	0.110 ± 0.005	10	30

### Nanoparticle uptake in mesothelioma and healthy mesothelium cell lines

**Figure 2:**
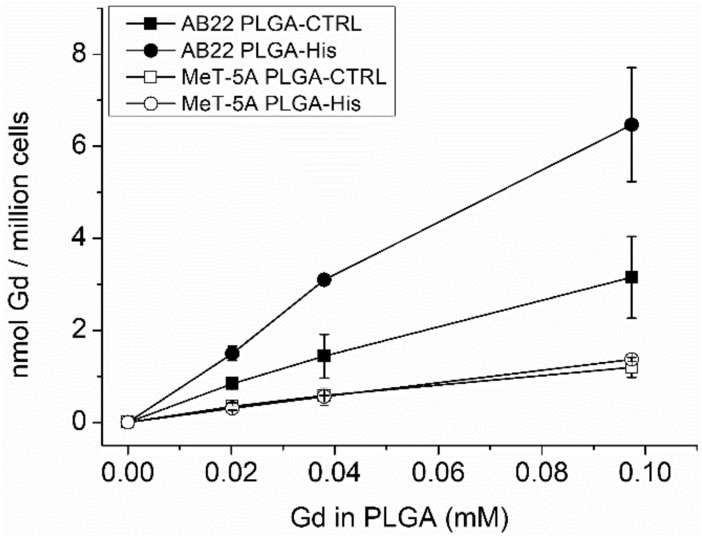
Uptake study performed incubating increasing Gd concentration in PLGA-CTRL and PLGA-His on AB22 and MeT-5A for 24 h at 37 °C, 5% CO_2_.

Uptake studies were performed using AB22 murine mesothelioma and, as a control, MeT-5A human healthy mesothelium cell lines, respectively. The two cell lines were incubated for 24 h with increasing Gd concentrations of both PLGA-His and PLGA-CTRL and after 24 h the amount of internalized Gd and B were measured by ICP-MS and normalized to the total cell proteins. Figure 2 shows that the nanoparticle uptake by AB22 tumour cells appears to be overall increased compared to the healthy mesothelial MeT-5A cell line. Moreover, the uptake of the oligo-His coated nanoparticles seems to be furtherly enhanced in mesothelioma cell line AB22 compared to the unconjugated nanoparticle. In conclusion, the presence of histidine in PLGA-His does not affect the uptake of the NPs in healthy cells suggesting a specific pH triggered mechanism occurring only in malignant cells, making these NPs suitable to specifically target this aggressive type of tumour cells.

From the data obtained by ICP-MS analysis, by converting mg of proteins in number of cells (1 mg of protein correspond to 1.7 and 6.3 million cells for AB22 and MeT-5A, respectively) and assuming that in the case of epithelial tumours (with a diameter ranging 15–20 μm), a density of ca. 10^8^ cells for cm^3^ is a reasonable number for these tumour tissues^42^, it was possible to calculate the maximum ppm of B internalised in cells, reported in Table 4.

**Table 4:** Maximum ppm of boron internalized in AB22 and MeT-5A after 24 h incubation in the presence of PLGA-CTRL or PLGA-His.

Cells	PLGA-CTRL (ppm)	PLGA-His (ppm)
AB22	30.6 ± 8.6	62.8 ± 12.0
MeT-5A	4.3 ± 0.8	5.0 ± 0.17

**Figure 3:**
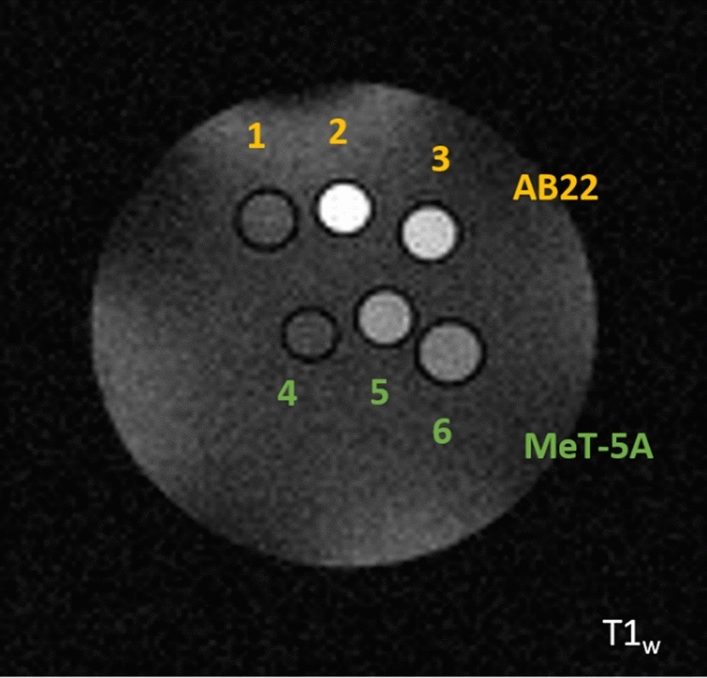
T_1w_ MRI image of glass capillaries containing AB22 and MeT-5A cell pellets incubated with PLGA-CTRL (3 and 6), PLGA-His (2 and 5) and untreated control cells (1 and 4).

Subsequently, in order to assess if the concentration of the internalized AT101 was sufficient for the generation of a detectable MRI Signal Intensity enhancement, a T_1_ weighted image was acquired on AB22 and MeT-5A cells incubated with PLGA-CTRL or PLGA-His, both at 0.097 mM (Gd concentration) and after washing, centrifuging the cells at the bottom of glass capillaries. As we can observe in Fig. 3, the uptake of the nanoparticles is consistent with the previous findings, suggesting an increased uptake of the PLGA-His in mesothelioma cells compared to healthy ones and also compared to the cells incubated with PLGA-CTRL.

### Neutron cell irradiation

**Figure 4:**
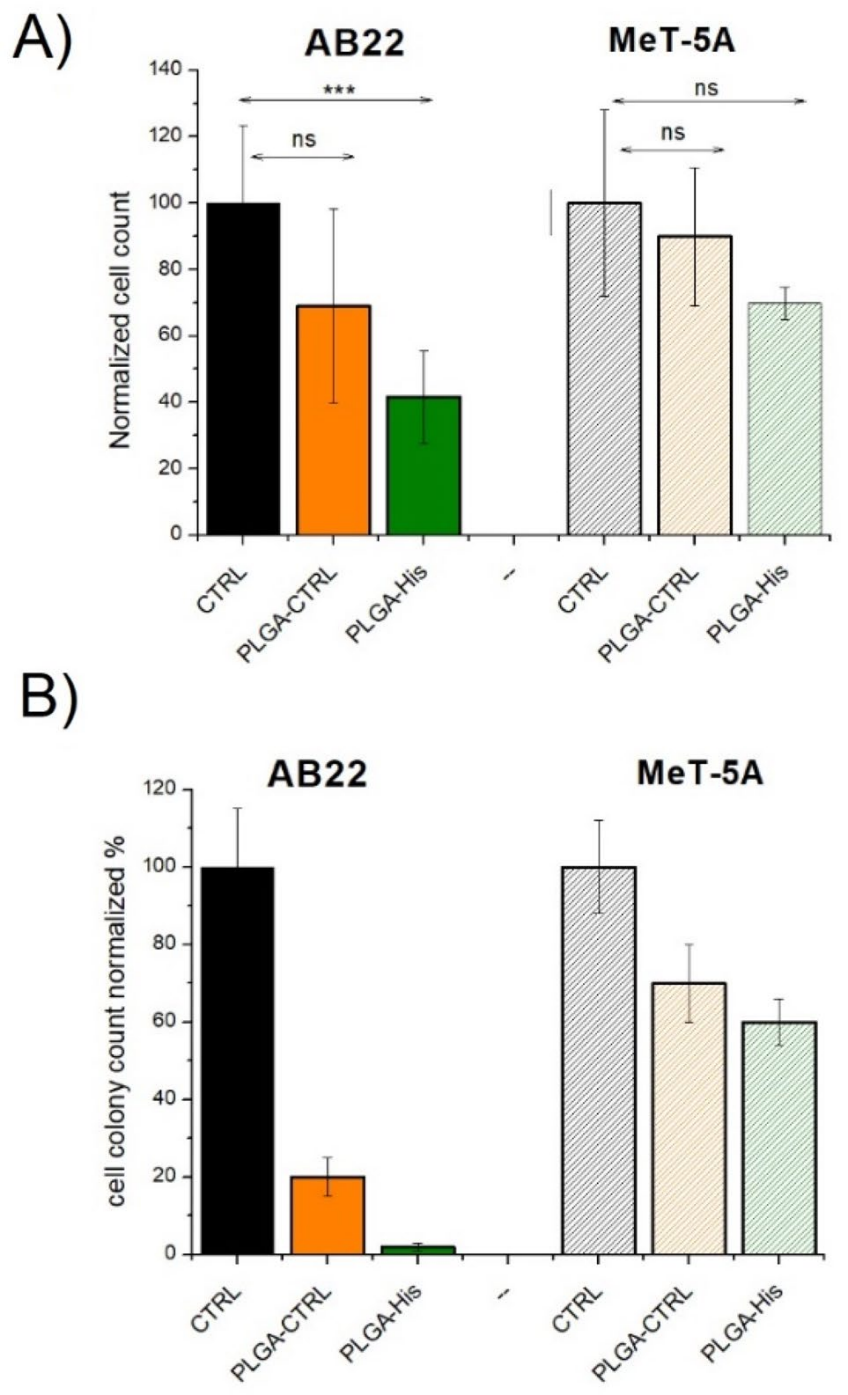
(**A**) Normalised cell count 24 h after neutron irradiation, performed on AB22 and MeT-5A previously treated with PLGA-CTRL or PLGA-His and compared to untreated cells. Statistical Significance was determined by Student T-test (***P < 0.01); (**B**) clonogenic assay performed 18 days after neutron irradiation. The number of colonies was calculated by ImageJ software. The % was calculated by setting the irradiated control cells to 100%.

Cells incubated with PLGA nanoparticles encapsulating AT101 were irradiated with thermal neutrons at the Triga Mark II nuclear reactor at the University of Pavia. For this set of experiments, AB22 and MeT-5A cells were pre-incubated for 24 h in three different conditions: (1) with medium only (CTRL cells), (2) with PLGA-CTRL and (3) with PLGA-His. PLGA-NPs were incubated at a concentration corresponding to a 0.097 mM Gd concentration in the cell medium. Cells were irradiated at the thermal column for 15′ with a reactor power of 30 kW. As shown in Fig. 4A, a strong cytotoxic effect of BNCT is clearly detected on AB22 cells, with a significant decrease in the number of cells 24 h after irradiation. In fact, after irradiation, compared to the irradiated control group, 30% of the AB22 cells treated with PLGA-CTRL died, and regarding PLGA-His, the percentage increases to 60%. On the contrary, MeT-5A cells showed no significant decrease 24 h after irradiation, consistent with the lower NPs uptake of this cell type (as shown in Fig. 2 and Table 2). This observation is supported by the results obtained with clonogenic assay carried out at day 18 after irradiation. In fact, Fig. 4B shows that AB22 after irradiation, in particular if treated with PLGA-His, stop to form colonies. These results suggest a significant decrease in the ability of mesothelioma cells to reattach and when treated with PLGA NPs, particularly effective in the case of PLGA-His administration. On the contrary, MeT-5A healthy cells show only a slightly reduced colony formation with respect to irradiated untreated control cells.

## Conclusions

Mesothelioma is an aggressive cancer that is associated with asbestos exposure. Although asbestos is banned in several countries, an epidemic of mesothelioma is predicted to affect middle-income countries during this century owing to their heavy consumption of asbestos. Patients affected by this disease often show a poor prognosis because of the lack of significant and effective therapies since conventional chemotherapy fails to affect and eradicate this cancer subtype effectively^43^. Although recent work has found significant possibilities for treatment, identifying genetic and pathophysiological vulnerabilities, mesothelioma remains one of the cancers with the lowest survival rate. Therefore, in this paper, we ought to demonstrate the possibility of an innovative approach, based on Boron Neutron Capture Therapy, with a theranostic compound delivered by PLGA nanoparticles. We developed and characterised PLGA nanoparticles containing poly-histidine chains, able to retain the theranostic compound AT101 effectively and deliver it specifically to the tumour cells, thus taking full advantage of this pre-targeted radiotherapy. It was demonstrated in this paper how these nanoparticles are preferentially taken-up by mesothelioma than healthy mesothelial cells, suggesting the possibility for a targeted therapeutic approach in the cure of this dreadful disease. Consequently, AB22 mesothelioma cells were strongly affected by neutron irradiation causing their irreversible damage, whereas a high percent of healthy cells was spared by the same treatment, as also demonstrated by the clonogenic assay. Therefore, we propose BNCT as a possible alternative therapeutic strategy to treat mesothelioma.

## Data Availability

The datasets used and analysed during the current study will be shared upon request. Requests should be addressed to the corresponding author.
